# The thermoregulatory role of relative bill and leg surface areas in a Mediterranean population of Great tit (*Parus major*)

**DOI:** 10.1002/ece3.8263

**Published:** 2021-11-03

**Authors:** Núria Playà‐Montmany, Erick González‐Medina, Julián Cabello‐Vergel, Manuel Parejo, José M. Abad‐Gómez, Juan M. Sánchez‐Guzmán, Auxiliadora Villegas, José A. Masero

**Affiliations:** ^1^ Conservation Biology Research Group Facultad de Ciencias Universidad de Extremadura Badajoz Spain; ^2^ Ecology in the Anthropocene Associated Unit CSIC‐UEX Facultad de Ciencias Universidad de Extremadura Badajoz Spain

**Keywords:** bill size, evaporative water loss, Great tit, leg size, metabolic rate, thermoregulation

## Abstract

There is growing evidence on the role of legs and bill as ‘thermal windows’ in birds coping with heat stress. However, there is a lack of empirical work examining the relationship between the relative bill and/or leg surface areas and key thermoregulatory traits such as the limits of the thermoneutral zone (TNZ) or the cooling efficiency at high temperatures. Here, we explored this relationship in a Mediterranean population of Great tit (*Parus major*) facing increasing thermal stress in its environment. The lower and upper critical limits of the TNZ were found to be 17.7 ± 1.6ºC and 34.5 ± 0.7°C, respectively, and the basal metabolic rate was 0.96 ± 0.12 ml O_2_ min^−1^ on average. The evaporative water loss (EWL) inflection point was established at 31.85 ± 0.27°C and was not significantly different from the value of the upper critical limit. No significant relationship was observed between the relative bill or tarsi size and TNZ critical limits, breadth, mass‐independent VO_2_, or mass‐independent EWL at any environmental temperature (from 10 to 40°C). However, Great tit males (but not females) with larger tarsi areas (a proxy of leg surface area) showed higher cooling efficiencies at 40°C. We found no support for the hypothesis that the bill surface area plays a significant role as a thermal window in Great tits, but the leg surface areas may play a role in males’ physiological responses to high temperatures. On the one hand, we argue that the studied population occupies habitats with available microclimates and fresh water for drinking during summer, so active heat dissipation by EWL might be favored instead of dry heat loss through the bill surface. Conversely, male dominance behaviors could imply a greater dependence on cutaneous EWL through the upper leg surfaces as a consequence of higher exposure to harsh environmental conditions than faced by females.

## Graphical abstract

Despite recent studies highlighting the role of relative bill and leg sizes on bird thermoregulation, their relationship with thermal physiology has been rarely studied under controlled conditions. We found no support for the hypothesis that bill size plays a significant role as a thermal window in a Mediterranean population of Great tit *Parus* *major* *,* but leg size could have a role on males’ physiological responses to high temperatures. We suggested that sex‐related differences in the role of leg surface in bird thermoregulation could be related to contrasting behavioral patterns.

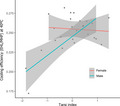

## 1 INTRODUCTION

The bill and legs of birds are multifunctional appendages that are involved in well‐known roles with direct fitness costs such as foraging or locomotion (e.g., Moreno & Carrascal, 1993; Tattersall et al., 2017). Both appendages also become key regions in body thermoregulation due to their contribution to heat and water balances, roles that have been emphasized by studies using thermographic images (e.g., Friedman et al., 2019; Greenberg et al., 2012; Tattersall et al., 2009). Illustrative examples are the Toco toucan *Ramphastos toco*, which can lose up to 60% of its total body heat through its bill (Tattersall et al., 2009), and herons and gulls, which increase their heat loss through their legs as the ambient temperature increases (Steen & Steen, 1965). This role of the bill and legs as ‘thermal windows’ relies on the vascular network located under the skin; this network allows body heat dissipation via radiation and convection (e.g., Hagan & Heath, 1980; Steen & Steen, 1965; Symonds & Tattersall, 2010; Tattersall et al., 2009). Due to the anatomical differences between the vascular systems providing blood supply to the legs and bill (a countercurrent system in the legs and a more random and ramified vascular arrangement in the bill; Arad et al., 1989; Hagan & Heath, 1980; Midtgård, 1981; Tattersall et al., 2012), the relative importance of each appendage in a bird's ability to cope with thermal stress may be different (Symonds & Tattersall, 2010; Tattersall et al., 2012; Winder et al., 2020). At 35°C, four species of Darwin's finches, for example, lost dry heat through their bills, while their legs became areas of heat gain (see Tattersall et al., 2018).

A growing number of studies have documented body size changes—including bill and leg lengths—across diverse bird taxa in response to global warming (e.g., Campbell‐Tennant et al., 2015; Gardner et al., 2011; Sheridan & Bickford, 2011; Yom‐Tov, 2001), but the underlying physiological mechanisms responsible for these changes remain poorly understood. According to ‘Allen's rule’ (Allen, 1877), birds from warm climates tend to have larger appendages relative to their body size than those from colder climates; this feature of birds from warm climates may favor body heat dissipation in hot environments. Thus, having relatively large unfeathered appendages could increase birds’ thermal tolerance (Gardner et al., 2016), which is becoming critically more necessary due to the increases in the frequency, severity, and duration of extreme heat events (McKechnie & Wolf, 2019; Stillman, 2019). In this context, interest in the role of bill and leg surface areas—especially bill size—as effective mechanisms of dry heat dissipation has increased in recent years (e.g., Gardner et al., 2016; Greenberg et al., 2012; Ryeland et al., 2017, 2019, 2021; Tattersall et al., 2009, 2017).

The thermal gradient between the environmental temperature (*T*_a_) and body temperature (*T*_b_) seems to be key in determining a bird's capacity to lose dry heat through the bill and leg appendages (Greenberg et al., 2012; Powers et al., 2017; Tattersall et al., 2009, 2017, 2018). When *T*_a_ is approximately equal to *T*_b_ (39–42°C; Bartholomew & Cade, 1963), the capacity of the bill to lose dry heat (i.e., its ability to work as a thermal radiator) starts to diminish (e.g., Powers et al., 2017; Tattersall et al., 2018). When *T*_a_ is greater than *T*_b_, the bill stops working as a thermal radiator and can turn into a heat input source (Gardner et al., 2016; Greenberg & Danner, 2012; Ryeland et al., 2017). Thus, maximum *T*_a_ has been suggested as a driver of variations in bill size (Greenberg & Danner, 2012; Greenberg et al., 2012). Despite this, there is a lack of empirical work examining the relationship between relative bill and leg sizes and metabolic heat production or evaporative heat loss within and outside bird's thermoneutral zone (TNZ), the range of *T*_a_ in which homeothermy can be maintained without additional costs in terms of energy or water. Further studies that delve into this relationship are required for a better understanding of thermoregulation in birds in the context of global warming.

As *T*_a_ decreases or increases below the lower (*T*_lc_) or above the upper (*T*_uc_) critical limits of the TNZ, respectively, energy expenditure increases to maintain homeothermy (McNab, 2002). *T*_uc_ is generally lower than *T*_b_, so if the bill and unfeathered leg surfaces work as thermal radiators, we should expect that the larger their relative surface areas are, the greater the loss of dry heat at *T*_a_ within the range between *T*_uc_ and *T*_b_, which in turn should lead to less energy consumption and lower evaporative water loss (EWL). The latter is the only avenue by which birds can maintain normothermic *T*_b_ when *T*_a_ exceeds *T*_b_ (e.g., McKechnie & Wolf, 2019) and is an energetically expensive process that is affected by the environmental humidity (e.g., Smith et al., 2015, van Dyk et al., 2019). This implies a critical trade‐off between the need to avoid hyperthermia and the risk of dehydration, particularly in bird species inhabiting hot and arid environments (Boyles et al., 2011; Czenze et al., 2020; Oswald et al., 2018; Smit et al., 2013; Song & Beissinger, 2020).

In this study, we calculated the TNZ of a Mediterranean population of Great tit (*Parus major*) (~16 g), a widespread and abundant passerine that is a well‐known ecological model used to investigate the relationships between appendage morphology and ecological traits (e.g., Gosler, 1987, 1993). We then examined the relationships between the relative bill and leg surface areas to body size and several thermoregulatory traits (the rate of O_2_ consumption [VO_2_], EWL, *T*_lc_, *T*_uc_, TNZ breadth, *T*_a_ inflection point of EWL, and evaporative cooling efficiency). We hypothesized that when *T*_a_ exceeds *T*_uc_ but is lower than *T*_b_, individuals with larger relative bill and/or leg sizes will consume less oxygen to maintain homeothermy, whereas when *T*_a_ is lower than *T*_lc_, these individuals will consume more oxygen to avoid hypothermia. We further predict a lower *T*_a_ inflection point of EWL in individuals with lower relative thermal appendage sizes.

## 2 MATERIALS AND METHODS

All procedures were approved by the bioethical committee of the University of Extremadura, Spain (108/2016) and were conducted under the governmental license CN0032/18/ACA.

### 2.1 Capture and biometric measurements

The Great tit individuals examined belonged to a population located in the areas surrounding the city of Badajoz (SW Spain; 38° 56′ 7.85″N, 6° 56′33.129″). Classified as Csa according to the Köppen Climatic Classification (mean annual *T*_a_: 17.27 ± 0.05°C and summer mean maximum *T*_a_: 34.18 ± 0.07°C; data from 1998 to 2018, State Meteorological Agency), this area has experienced a significant increase in the summer maximum *T*_a_ and frequency and duration of heat waves over the last three decades (Acero et al., 2014, 2018). A total of 24 Great tits were collected as nestlings and hand‐raised in the laboratory of the University of Extremadura during spring 2017. Individuals were maintained in artificial nests where they were fed every 2 h from 8:00 a.m. to 10 p.m. until fledging. When birds were completely independent, they were individually identified with an alphanumeric band and moved to outdoor aviaries (5 m × 2.5 m × 2 m each) equipped with small ponds with running water, natural vegetation, and live prey where they stayed during several months before metabolic trials started (winter 2019). Taking advantage of a parallel study, we added data from eight wild‐living individuals (five juveniles and three adults) caught in the same study area to optimize our sample size of individuals representing each sex. These Great tits were captured in the wild by mist‐nets in the late afternoon, measured at night (winter 2019), and released early the next morning.

The age and sex of the wild‐living birds were determined according to their plumage characteristics (Svensson, 1992), and the sex of all individuals (16 males and 16 females) was later confirmed by CHD‐based molecular sexing protocols (Griffiths et al., 1998). Hand‐raised birds were released at their place of collection several weeks after respirometry measurements.

Bill and tarsi surface areas were estimated individually following Greenberg et al. (2012). Briefly, we used an equation in which the bill area is approximated to an elliptical cone:

$$\left(\frac{\text{BW}+\text{BD}}{4}\right)\text{BL}\times \pi$$

where BW is the bill width, BD is the bill depth, and BL is the bill length (see figure in Svensson, 1992).

Measurements of the tarsus were used to estimate the tarsi surface area as a proxy of the leg surface area using the equation for an elliptical cylinder:

$$\left(\pi {\left(2\left({\left(\frac{\text{TW}}{2}\right)}^{2}+{\left(\frac{\text{TD}}{2}\right)}^{2}\right)‐0.5{\left(\text{TW}‐\text{TD}\right)}^{2}\right)}^{\frac{1}{2}}\right)\text{TL}\times 2$$

where TW is the tarsus width, TD is the tarsus depth (both measured at the midpoint of the tarsus), and TL is the tarsus length. All bill and tarsus measurements were performed by the same person (JMAG) using a digital caliper (± 0.01 mm).

We also measured the wing length (flattened and straightened) as a proxy of body size using a wing rule (± 0.5 mm; Gosler et al., 1998).

### 2.2 Gas exchange measurements

We measured O_2_ consumption (ml/min) and EWL (mg/h) using an open flow‐through respirometry system. Each individual was placed in a polypropylene metabolic chamber (232 × 165 × 162 mm; effective volume = 3.9 L), the floor of which was covered with a 1 cm mineral oil layer to avoid evaporation from excreta. The chambers were equipped with a wire mesh platform located 3 cm above this oil layer to allow individuals to perch without touching the oil. All metabolic chambers were placed in a temperature‐controlled cabinet (ICP, 750 Memmert GmbH), where the increasing or decreasing *T*_a_ profiles (see below for details) were created automatically using control software. We introduced a calibrated thermistor probe (±0.001°C) inside the metabolic chambers to monitor the *T*_a_ during the metabolic trials. Exterior dry air (<1 kPa WVP) was pumped from an air dryer compressor (MESTRA^®^) into a carboy (Lighton, 2008) and then directed to the metabolic chambers using mass flow controllers (MFS, Sable Systems International). Flow rates of 1000 or 3000 ml/min (depending on data collection protocol; see details below) were used during metabolic trials. Excurrent airstreams from the chambers flowed through an eight‐channel multiplexer (RM‐8, Sable Systems International), which automatically alternated every 360 s between metabolic chambers containing birds as well as an additional chamber left empty to obtain baseline values. The latter were obtained for 300 s at the start of every trial and following two metabolic chamber measurements. We subsampled the downstream air at 200 ml/min (SS3 subsampler, Sable Systems International) and pulled it sequentially through an H_2_O analyzer (RH300, Sable Systems), a Drierite^®^ column, and an O_2_ analyzer (FC‐10 Oxygen Analyzer, Sable Systems). The data were digitalized using an analog‐to‐digital converter (UI2 model, Sable Systems) and recorded with a sampling interval of 1 s using Expedata software (version 1.9.14, Sable Systems). Both analyzers were zeroed and spanned weekly using standard protocols (Lighton, 2008).

### 2.3 Data collection protocol

Gas exchange rates were measured through a wide range of *T*_a_s (10, 15, 20, 25, 30, 35, 37, and 40°C) in a stepped manner in a maximum of six individuals at a time. The metabolic trials were performed at night (from 8:00 p.m. to 8:00 a.m.; the daily resting phase of Great tit) after the food was withheld from the birds for at least 2 h to ensure they were in a postabsorptive state (RER 0.70). To determine *T*_lc_, six birds at a time were exposed alternately to an increasing or a decreasing stepped *T*_a_ profile ranging from 10 to 30°C or vice versa. All individuals were exposed to each *T*_a_ for a minimum of 65 min using a flow rate of 1000 ml/min. For *T*_uc_ determination, individuals were exposed to an increasing profile of *T*_a_s (35, 37, and 40°C). We used a flow rate of 3000 ml/min to ensure maintenance of low humidity levels (<1 kPa WVP), which aided in keeping birds calm (Whitfield et al., 2015), and only two birds were measured per trial; they were exposed to each *T*_a_ for a minimum of 25 min. The first 65 min or 25 of the stepped *T*_a_ profiles of each protocol were used to ensure that the individuals were acclimated to the metabolic chambers (i.e., stable VO_2_ and EWL traces) after handling. To ensure captive individuals recovered from the stress of handling following *T*_lc_ measurement, bird exposition to the highest *T*_a_s (35, 37, and 40°C) was conducted after 2 weeks. Individual's behavior within chambers was monitored directly by an observer (we did not record videos) using infrared cameras to ensure they remained calm during the metabolic measurements. All individuals were hydrated and weighed (±0.1 g) before and after the metabolic measurements. The mean body mass (*M*_b_) of the birds was used in the analyses.

### 2.4 Data analysis

The VO_2_ and EWL values at each *T*_a_ were estimated as the lowest stable 2‐min (see, for example, Boratyński et al., 2016) values using Eqs. 10.2 and 10.9 from Lighton (2008), respectively, with a custom macro designed in Expedata. We used a respiratory quotient of 0.70 (e.g., Kvist & Lindström, 2001). To obtain the metabolic heat production (MHP), we converted the VO_2_ values to metabolic rates (Watt, W) using an energy equivalent of 20 kJ/L O_2_ (e.g., Caro & Visser, 2009). The drift of water and O_2_ traces was corrected using the Catmull‐Rom spline correction applied to baselines. The evaporative heat loss (EHL) was calculated assuming latent heat of vaporization values for water at 35, 37, and 40°C following Tracy et al. (1980). The evaporative cooling efficiency (EHL/MHP) was calculated at every *T*_a_ above *T*_uc_.

We used a generalized estimating equations (GEE) approach to simultaneously identify population limits of TNZ (*T*_lc_ and *T*_uc_) of our Great tit population (*n* = 24) using the ‘lme4’ package (Bates et al., 2014), the ‘geepack’ package (Halekoh et al., 2006), and a modified version of the ‘segmented’ package (Muggeo), in R 3.6.1. Then, we calculated *T*_lc_ and *T*_uc_ for each focal individual using the R packages ‘lme4’ and the modified version of ‘segmented’. We could not obtain TNZ values for wild‐living individuals since they were only measured under one of both protocols. The VO_2_ values were corrected by body mass using residuals from a regression between VO_2_ and body mass (log‐transformed values). The TNZ breadth was calculated as the *T*_uc_ value minus the *T*_lc_ value. Mean value of VO_2_ within TNZ was considered to be the basal metabolic rate (BMR). The inflection point of EWL was also calculated using ‘lme4’ and ‘segmented’ packages in R.

To obtain the relative appendage sizes (bill and tarsi index values), we computed the residuals of the regression of the bill or tarsi surface area on the wing length, as this is assumed to be the best proxy of body size in small‐sized passerines, including Great tits (Gardner et al., 2016; Gosler et al., 1998). We log‐transformed the variables to meet the assumptions of linearity, homoscedasticity, and normality. The residuals were calculated separately for males and females due to sexual dimorphism in size. Great tit males had larger wing lengths (*t*_30_ = −2.27, *p* < .05), higher *M*_b_ values (*t*_30_ = −2.38, *p* < .05), and bill surface areas (*t*_30_ = −2.16, *p* < .05) than females, but the sexes did not differ in tarsi surface area (*t*_30_ = −1.17, *p* = .25). We calculated mass‐independent VO_2_ and mass‐independent EWL from the regressions of the VO_2_ and EWL rates, respectively, on the mean *M*_b_.

To ensure that the order of exposure of each individual to *T*_a_ did not affect the metabolic measurements from 10 to 30°C, we performed a *t*‐test to compare the mass‐independent VO_2_ and mass‐independent EWL values between individuals measured at the decreasing or increasing stepped *T*_a_ profiles. No significant differences were found in the analysis (all results *p* > .09), so the order of *T*_a_ exposure was not considered in the models.

To test the effects of the bill and tarsi indices on physiological traits (*T*_lc_, *T*_uc_, TNZ breadth, the *T*_a_ inflection point of EWL, EHL/MHP, and the mass‐independent VO_2_ and mass‐independent EWL at each *T*_a_), we built a series of generalized linear models that included physiological traits as response variables, sex (two levels) as a fixed factor, bill and tarsi indices as covariates, and the interactions between the bill index and sex, and between the tarsi index and sex, as fixed factors. In the case of EHL/MHP, *M*_b_ was included as a covariate. Multicollinearity was tested by calculating the variance inflation factor (VIF) among all predictor variables using the ‘car’ package (Fox & Weisberg, 2019); we confirmed no collinearity problems (all VIF values <5; see Zuur et al., 2010). The model selection was based on the Akaike information criterion for small sample sizes (AIC_c_) to identify the top model(s) (models within 2 ΔAICc of the top model), and the AICc weights (*w*_i_) were used to further distinguish among the top models (Burnham & Anderson, 2002). We used the function ‘dredge’ from the R package MuMIn (Barton, 2018) for this procedure.

In cases where more than one model had ΔAICc <2 but *w*_i_ <0.9 (Burnham & Anderson, 2002), we performed model averaging (Grueber et al., 2011). A predictor was considered significant when the 95% confidence interval (CI) for the estimated coefficient did not overlap zero. We further calculated the relative importance weight (RIW) of each explanatory variable (see Table 2).

Statistical analyses were conducted in SPSS Statistics 23 (SPSS Inc.) and R 4.0.3 (R Core Team, 2014), and figures were produced using the R package ‘ggplot2’ (Wickham, 2016). Values are shown as means ± SEs.

## 3 RESULTS

**FIGURE 1:**
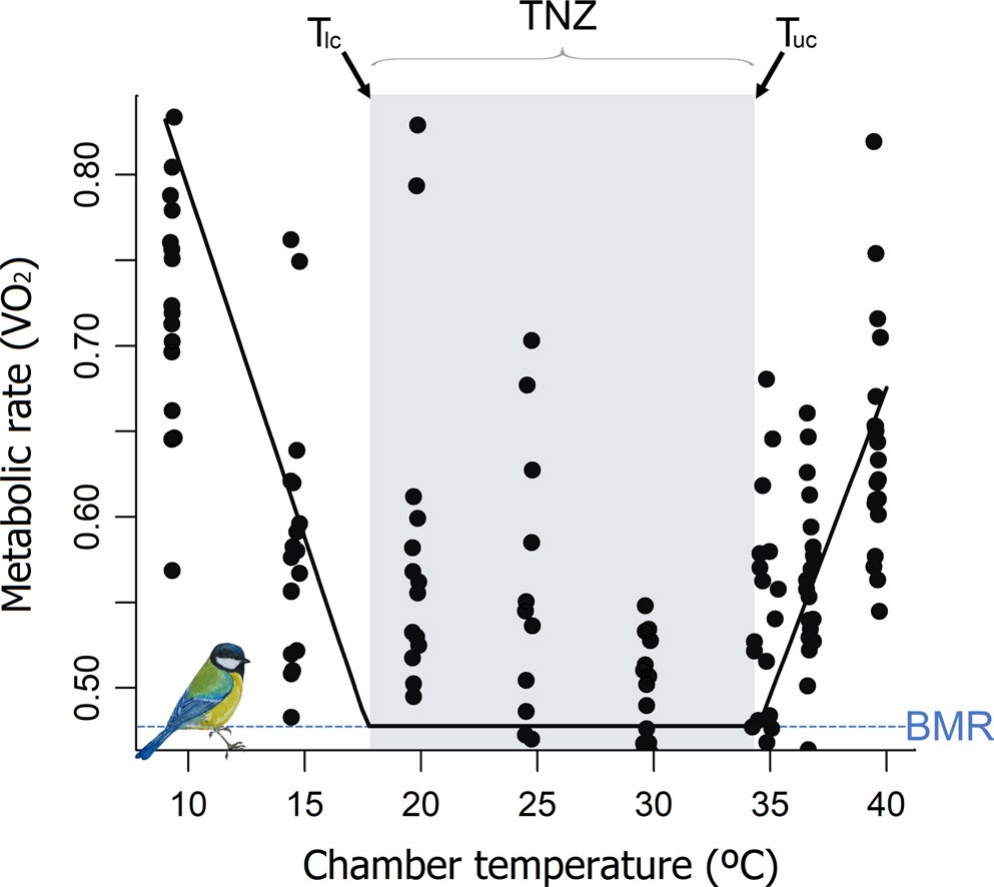
The relationship between metabolic rate (VO_2_) (measured as mass‐corrected oxygen consumption) and the environmental temperature (*T*_a_) is mainly represented as a U‐shape curve where the thermoneutral zone (TNZ) is delimited by the lower (*T*_lc_) and upper (*T*_uc_) critical temperatures. The TNZ of our Great tit population (*n* = 24) was measured during the rest phase of the species. Each point represents a measurement for one individual. The lowest inflection point corresponds to the *T*_lc_ (17.7 ± 1.63°C) and the highest corresponds to the *T*_uc_ (34.5 ± 0.71°C). The breath TNZ of our population was 16.8 ± 1.17°C

**FIGURE 2:**
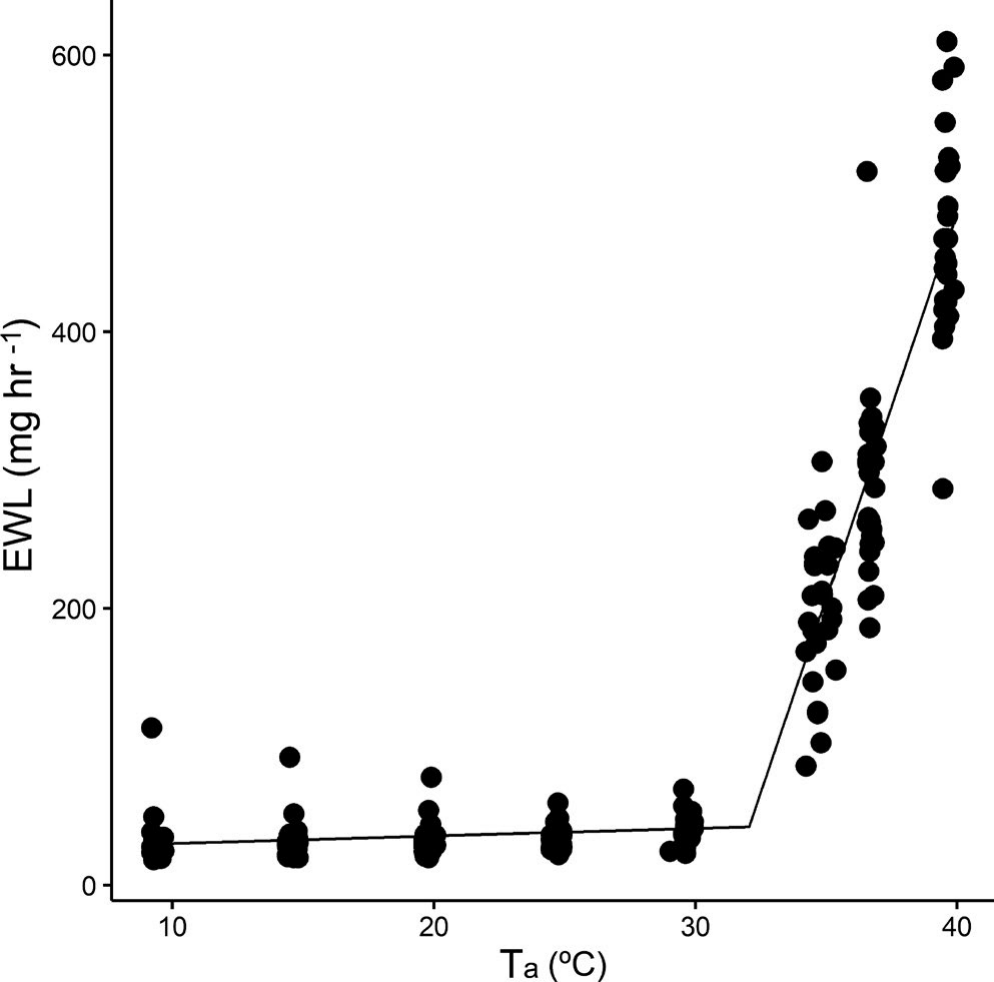
Evaporative water loss (EWL) in a Mediterranean population of Great tits (*n* = 24) from 10 to 40°C

The estimated BMR was 0.96 ± 0.12 O_2_ ml min^−1^, and the TNZ breadth, *T*_lc_, and *T*_uc_ were 16.8 ± 1.2, 17.7 ± 1.6, and 34.5 ± 0.7°C, respectively (Figure 1). The EWL inflection point was established at 31.85 ± 0.27°C (Figure 2) and was not significantly different from the *T*_uc_ value (*t*_23_ = −1.37, *p* = .18).

The null model emerged as the top‐ranked model for most of the variables analyzed (see Table 1). There was no detectable relationship between any of the physiological measures and either relative bill or relative tarsus surface area at any *T*_a_ despite these indices were included in the best models (95% CIs overlapped zero in all cases; Tables 1 and 2). The null model was the best‐fitting model for the mass‐independent VO_2_ at 10, 15, and 35°C and the mass‐independent EWL at 40°C (Table 1).

**TABLE 1:** Top‐ranked candidate models explaining thermoregulatory traits in a Mediterranean population of Great tits in winter, including lower critical temperature (*T*_lc_), upper critical temperature (*T*_uc_), thermoneutral zone (TNZ) breadth, cooling efficiency (EHL/MHP), oxygen consumption (VO_2_), basal metabolic rate (BMR), and evaporative water loss (EWL)

Model	*df*	logLik	AICc	ΔAICc	*w*_i_	*R* ^2^
*T*_lc_						
Null	2	16.39	−28.20	0.00	0.26	.00
Sex	3	17.57	−27.90	0.27	0.23	.09
Bill index	3	17.03	−26.90	1.35	0.13	.05
Bill index + sex	4	18.46	−26.80	1.41	0.13	.16
*T*_uc_						
Null	2	36.28	−68.00	0.00	0.34	.00
Sex	3	37.32	−67.40	0.55	0.55	.08
Inflection *T*_a_ of EWL						
Null	2	42.69	−80.80	0.00	0.37	.00
Sex	3	43.32	−79.40	1.33	0.19	.05
TNZ breadth						
Bill index	3	22.32	−37.40	0.00	0.28	.13
Null	2	20.71	−36.90	0.59	0.21	.00
Bill index + Sex + Bill index × Sex	5	24.79	−36.20	1.20	0.15	.29
EHL/MHP at 35°C						
Tarsi index	3	53.43	−99.80	0.00	0.31	.13
Null	2	51.56	−98.60	1.18	0.17	.00
EHL/MHP at 37°C						
Tarsi index	3	56.10	−105.10	0.00	0.24	.13
Null	2	54.33	−104.10	0.98	0.15	.00
EHL/MHP at 40°C						
Tarsi index + Sex + Tarsi index × Sex	5	69.97	−126.90	0.00	0.58	.51
Mass‐independent VO_2_ at 10°C						
Null	2	−39.00	82.50	0.00	0.45	.00
Mass‐independent VO_2_ at 15°C						
Null	2	−39.00	82.50	0.00	0.46	.00
Mass‐independent BMR						
Null	2	−39.00	82.50	0.00	0.38	.00
Bill index	3	−38.60	84.10	1.69	0.16	.03
Tarsi index	3	−38.63	84.20	1.77	0.16	.02
Mass‐independent VO_2_ at 35°C						
Null	2	−34.74	74.00	0.00	0.45	.00
Mass‐independent VO_2_ at 37°C						
Null	2	−35.15	74.80	0.00	0.26	.00
Tarsi index	3	−34.25	75.60	0.78	0.18	.07
Bill index + Tarsi index	4	−33.06	76.00	1.19	0.14	.15
Bill index	3	−34.47	76.00	1.21	0.14	.05
Mass‐independent VO_2_ at 40°C						
Null	2	−34.62	73.80	0.00	0.38	.00
Tarsi index	3	−33.96	75.00	1.25	0.20	.05
Mass‐independent EWL at 10°C						
Null	2	−39.00	82.50	0.00	0.43	.00
Bill index	3	−38.69	84.30	1.89	0.17	.02
Mass‐independent EWL at 15°C						
Null	2	−39.00	82.50	0.00	0.37	.00
Bill index	3	−38.41	83.80	1.32	0.19	.04
Mass‐independent EWL at TNZ						
Null	2	−39.00	82.50	0.00	0.35	.00
Bill index	3	−38.16	83.30	0.82	0.23	.06
Mass‐independent EWL at 35°C						
Tarsi index	3	−31.91	70.9	0.00	0.45	.20
Mass‐independent EWL at 37°C						
Null	2	−34.74	74.00	0.00	0.39	.00
Tarsi index	3	−34.11	75.30	1.31	0.20	.05
Mass‐independent EWL at 40°C						
Null	2	−31.26	67.0	0.00	0.44	.00

Note

Models were selected using the Akaike Information Criterion with a correction for small samples (AICc). Only models with ΔAICc <2 are shown.

**FIGURE 3:**
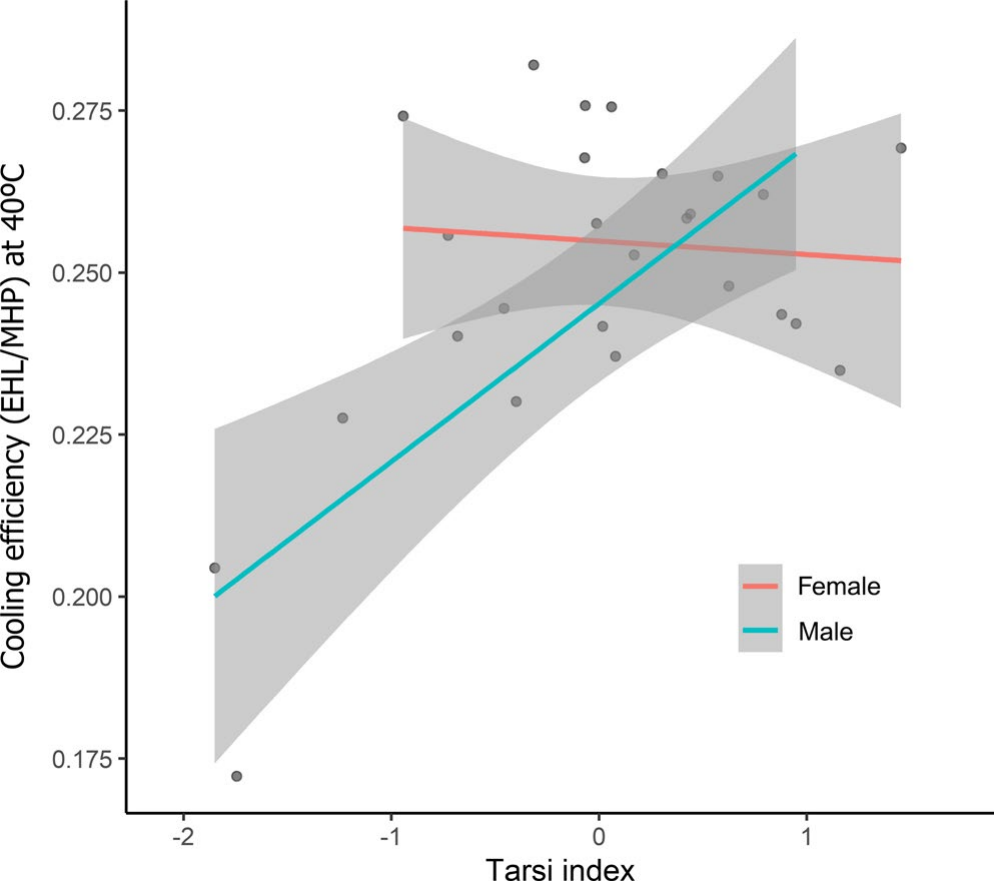
Evaporative cooling efficiency (EHL/MHP) in relation to tarsi index at 40°C in Great tits (log‐transformed values)

Only in the case of the EHL/MHP value at 40°C did we find a potential role of the leg surface area, as the interaction between the tarsi index and sex was included in the best model and was significant (Tables 1 and 2). Males with larger leg areas showed higher cooling efficiencies at 40°C (*F*_1,10_ = 17.82, *p* < .05), but this relationship was not found to be significant in females (*F*_1,12_ = 0.10, *p* = .76; Figure 3).

**TABLE 2:** Model‐averaged parameter estimates (±SE) for predictors of thermoregulatory traits in Great tits including lower critical temperature (*T*_lc_), upper critical temperature (*T*_uc_), thermoneutral zone (TNZ) breadth, cooling efficiency (EHL/MHP), oxygen consumption (VO_2_), basal metabolic rate (BMR), and evaporative water loss (EWL)

Model	Estimate	CI (95%)	SE	RIW
*T*_lc_				
Intercept	1.24	–	0.03	–
Bill index	0.01	−0.03, 0.11	0.03	0.35
Sex M	−0.04	−0.18, 0.03	0.05	0.48
*T*_uc_				
Intercept	1.54	–	0.01	–
Sex M	−0.01	−0.08, 0.01	0.02	0.43
*T*_a_ inflection of EWL				
Intercept	1.52	–	0.01	–
Sex M	−0.01	−0.05, 0.02	0.01	0.34
TNZ breadth				
Intercept	1.22	–	0.02	–
Bill index	−0.04	−0.13, 0.01	0.04	0.68
Sex M	0.01	−0.05, 0.11	0.02	0.24
Bill index × Sex M	0.03	−0.00, 0.23	0.06	0.24
EHL/MHP at 35°C				
Intercept	0.14	–	0.01	–
Tarsi index	0.01	−0.00, 0.03	0.01	0.64
EHL/MHP at 37°C				
Intercept	0.19	–	0.01	–
Tarsi index	0.01	−0.00, 0.03	0.01	0.62
EHL/MHP at 40°C				
Intercept	0.25	–	0.00	–
Tarsi index	−0.00	−0.02, 0.01	0.01	–
Sex M	−0.01	−0.02, 0.00	0.01	–
Tarsi index × sex M	0.03	0.01, 0.04	0.01	–
Mass‐independent VO_2_ at 10°C				
Intercept	−0.00	–	0.18	–
Mass‐independent VO_2_ at 15°C				
Intercept	−0.00	–	0.18	–
Mass‐independent BMR				
Intercept	−0.00	–	0.18	–
Bill index	0.04	−0.22, 0.55	0.12	0.23
Tarsi index	0.04	−0.25, 0.60	0.13	0.22
Mass‐independent VO_2_ at 35°C				
Intercept	−0.04	–	0.18	–
Mass‐independent VO_2_ at 37°C				
Intercept	−0.01	–	0.20	–
Bill index	0.13	−0.20, 0.86	0.23	0.40
Tarsi index	−0.15	−0.80, 0.14	0.23	0.44
Mass‐independent VO_2_ at 40°C				
Intercept	0.04	–	0.19	–
Tarsi index	−0.09	−0.71, 0.21	0.18	0.35
Mass‐independent EWL at 10°C				
Intercept	−0.00	–	0.18	–
Bill index	−0.04	−0.52, 0.24	0.12	0.28
Mass‐independent EWL at 15°C				
Intercept	−0.00	–	0.18	–
Bill index	−0.07	−0.57, 0.18	0.15	0.34
Mass‐independent EWL at TNZ				
Intercept	0.00	–	0.18	–
Bill index	0.09	−0.14, 0.61	0.17	0.40
Mass‐independent EWL at 35°C				
Intercept	−0.04	–	0.19	–
Tarsi index	−0.12	−0.57, 033	0.18	0.34
Mass‐independent EWL at 37°C				
Intercept	0.04	–	0.19	–
Tarsi index	0.08	−0.22, 0.71	0.18	0.34
Mass‐independent EWL at 40°C				
Intercept	0.04	–	0.18	–

Note

The 95% confidence intervals (CI) and relative importance of the parameters (RIW) are also shown.

## 4 DISCUSSION

We characterized some main thermoregulatory traits, such as TNZ breath and cooling efficiency at temperatures above *T*_uc_, in a Mediterranean Great tit population. We found no evidence for the hypothesis that the bill surface area plays a significant role as a thermal window to maintain normothermia. However, the tarsi surface area—a proxy of the leg surface area—could play a relevant role in males’ physiological responses to high *T*_a_s in the studied Great tit population.

Populations exposed to higher *T*_a_ values are expected to have higher critical thermal limits (e.g., Cooper & Swanson, 1994; Nilsson et al., 2016). Our findings support this statement, as the studied Mediterranean Great tit population showed a TNZ with a higher upper critical limit (6°C higher) than Great tits from cold environments in northern Europe (see Broggi et al., 2005). Nevertheless, data estimated for Great tits from Russia (Gavrilov, 2014) showed similar values to those found in our research (*T*_lc_: 17.7°C; *T*_uc_: 34.5°C), but these data must be taken with caution since the methodology used in that study to calculate the TNZ values differed from our methods and those used by other authors.

A clear link was obtained between the onset of EWL and the *T*_uc_, highlighting the importance of EWL as the main mechanism of heat loss when *T*_a_ surpasses *T*_uc_ (Wolf & Walsberg, 1996). The inflection point of EWL in Great tits contrasts with those observed in similar‐sized passerines acclimated to hot and arid climates, such as the Yellow‐plumed honeyeater *Ptilotula arnata* (~16 g) or the House finch *Haemorhous mexicanus* (~18 g), which showed EWL inflection points of 38°C (McKechnie et al., 2017, Smith et al., 2017). Nonetheless, it was similar to the ~35°C threshold seen in the Cape rockjumper *Chaetops frenatus* inhabiting a Mediterranean climate (Oswald et al., 2018). The need to use EWL as a thermoregulatory physiological mechanism may be extremely dangerous in passerines living in hot and arid zones such as deserts (McKechnie et al., 2021). However, forest species such as Great tits, even those inhabiting hot environments, would be subject to lower trade‐offs between dehydration and hyperthermia avoidance because they normally occupy buffered habitats during summer with available microclimates and fresh water for drinking, resulting in a lower EWL inflection point. Drinking water also allows higher EWL scopes, facilitating greater body heat loss effectiveness (Czenze et al., 2020). Thus, in habitats where water is accessible and exposure to solar radiation can be avoided by microhabitat selection, active heat dissipation might be favored in small passerines such as Great tits instead of losing body heat through passive pathways such as radiation (Greenberg et al., 2012). This could explain why, contrary to our predictions, we did not find evidence of a significant effect of the bill as a thermal window. Moreover, in our Great tit population, the bill and leg surface area are only about 1% and 3% of the whole‐body surface area, respectively (calculations performed following to Walsberg & King, 1978). Therefore, bill surface area clearly represents only a small part of the whole‐body surface, so its absolute role might be so minor that the effect size is undetectable.

Thermoregulatory behaviors reduce the need to engage in costly physiological responses (e.g., Amat & Masero, 2004; Angilletta, 2009; Dawson, 1982; Oswald et al., 2019; Thompson et al., 2018). For example, passerine species such as the Cape rockjumper *Chaetops frenatus* or the Rufous‐eared warbler *Malcorus pectoralis* increased their cool microsite use at higher *T*_a_ values (Oswald et al., 2019; Pattinson & Smit, 2017). The behavioral mechanisms regulating *T*_b_ in the studied Great tit population have never been investigated systematically, but this population must exploit the thermal heterogeneity in their environment by selecting microhabitats with favorable *T*_a_s. We also observed, for example, Great tits adopting wing drooping inside the metabolic chambers at ~34°C (NPM pers. obs.), which occasionally matched the onset of panting. This behavior was also observed in Zebra finch (*Taeniopygia guttata*) individuals inside metabolic chambers when exposed to 40°C, favoring an increase in cutaneous evaporative heat loss (CEWL; Wojciechowski et al., 2021). However, during the metabolic trials, neither *T*_b_ nor behaviors were registered, impeding formal analyses to evaluate their possible effects on Great tit thermoregulation.

The Great tit's maximum cooling efficiency, 0.83 ± 0.06, was in accordance with previous values reported for passerines (Bartholomew et al., 1968; McKechnie et al., 2017; Smith et al., 2015; Whitfield et al., 2015) for which the maximum EHL/MHP value was <2. At high *T*_a_ (40°C), Great tit males displayed a positive relationship between the EHL/MHP ratio and tarsi surface index. When partitioning EWL between its respiratory and cutaneous components, passerines rely mainly on respiratory EWL to deal with heat stress, but CEWL can also contribute significantly to reducing *T*_b_ in such circumstances (Wolf & Walsberg, 1996; Wojciechowski et al., 2021). For example, in Verdin (*Auriparus flaviceps*), a small passerine, CEWL at 50°C can be up to three times higher than at 30°C (Wolf & Walsberg, 1996). The skin of the lower legs (tarsometatarsus and feet) of Great tits is not permeable to water, so CEWL through these surfaces is not possible (Bernstein, 1974; Martineau & Larochelle, 1988). However, CEWL could occur (in addition to other feathered parts of the skin) in the sparsely feathered surface of the upper legs, which could explain the higher EHL/MHP values recorded at 40°C in males with higher tarsi surface index values. The sex‐specific differences observed in this relationship could be related to the differences in behavioral patterns previously observed between the sexes in this species; males have been observed to sing and fight for broader periods of time to defend their mates and territories (Hindle, 1952). This male dominance behavior is related to increased exposure to harsh environmental conditions, and so having larger legs (and thus greater rates of CEWL) may aid males in maintaining normothermia. Sex‐related behaviors have previously been related to sex‐specific differences in the *T*_a_ threshold regarding the onset of heat dissipation behaviors (Kemp et al., 2020), which may also induce biased sexual selection over the next few decades (Miller et al., 2018). For example, in the nonpasserine Southern yellow‐billed hornbill (*Tockus leucomelas*) species, males and females have been observed showing different EWLs and RMRs at high *T*_a_ values, which is probably related to the contrasting behaviors displayed by the sexes during the breeding season (Van Jaarsveld et al., 2021). Future studies are needed to identify and improve our knowledge about the mechanisms that underlie these sex‐specific relationships.

Overall, our study provides an improved understanding of the thermal biology of a Mediterranean population of Great tits and shows the complex interplays that may exist between the relative sizes of the unfeathered appendages and the physiological traits involved in thermoregulation. Similar studies developed with birds that occupy poorly climatically buffered habitats (i.e., habitats with reduced microclimates to escape direct solar radiation or with limited availability of freshwater) could provide clarification on the roles of the bill and legs in the thermal physiology of Mediterranean bird populations. These would also improve our ability to predict the vulnerability of these birds to global warming and extreme heat events.

## CONFLICT OF INTEREST

The authors declare no conflicts of interest.

## AUTHOR CONTRIBUTION

**Núria Playà‐Montmany:** Conceptualization (equal); Data curation (equal); Formal analysis (equal); Investigation (equal); Methodology (equal); Software (equal); Validation (equal); Visualization (equal); Writing‐original draft (lead); Writing‐review & editing (equal). **Erick González‐Medina:** Data curation (equal); Formal analysis (equal); Visualization (equal); Writing‐review & editing (equal). **Julián Cabello‐Vergel:** Data curation (equal); Writing‐review & editing (equal). **Manuel Parejo:** Data curation (equal); Writing‐review & editing (equal). **José M. Abad‐Gómez:** Data curation (equal); Writing‐review & editing (equal). **Juan M. Sánchez‐Guzmán:** Data curation (equal); Funding acquisition (equal); Resources (equal); Writing‐review & editing (equal). **Auxiliadora Villegas:** Conceptualization (equal); Data curation (equal); Funding acquisition (equal); Investigation (equal); Methodology (equal); Project administration (equal); Resources (equal); Software (equal); Supervision (equal); Validation (equal); Writing‐original draft (supporting); Writing‐review & editing (equal). **José A. Masero:** Conceptualization (equal); Data curation (equal); Funding acquisition (equal); Investigation (equal); Methodology (equal); Project administration (equal); Resources (equal); Software (equal); Supervision (equal); Validation (equal); Writing‐original draft (supporting); Writing‐review & editing (equal).

## Data Availability

The dataset is available in the Dryad public repository: https://doi.org/10.5061/dryad.cc2fqz66z.
